# Dietary Astaxanthin Can Promote the Growth and Motivate Lipid Metabolism by Improving Antioxidant Properties for Swimming Crab, *Portunus trituberculatus*

**DOI:** 10.3390/antiox13050522

**Published:** 2024-04-26

**Authors:** Yao Deng, Shichao Xie, Wenhao Zhan, Hongyu Peng, Haiqing Cao, Zheng Tang, Yinqiu Tian, Tingting Zhu, Min Jin, Qicun Zhou

**Affiliations:** 1 Laboratory of Fish and Shellfish Nutrition, School of Marine Sciences, Ningbo University, Ningbo 315211, China; 2201130061@nbu.edu.cn (Y.D.); 2201130083@nbu.edu.cn (S.X.); 2201130085@nbu.edu.cn (W.Z.); 2201130074@nbu.edu.cn (H.P.); 2211130078@nbu.edu.cn (H.C.); 2211130111@nbu.edu.cn (Z.T.); 2211130113@nbu.edu.cn (Y.T.); 2001130080@nbu.edu.cn (T.Z.); 2 Key Laboratory of Green Mariculture (Co-Construction by Ministry and Province), Ministry of Agriculture and Rural, Ningbo 315211, China

**Keywords:** astaxanthin, growth performance, *Portunus trituberculatus*, antioxidant capacity, lipid metabolism

## Abstract

This study aimed to assess the influence of varying dietary levels of astaxanthin (AST) on the growth, antioxidant capacity and lipid metabolism of juvenile swimming crabs. Six diets were formulated to contain different AST levels, and the analyzed concentration of AST in experimental diets were 0, 24.2, 45.8, 72.4, 94.2 and 195.0 mg kg^−1^, respectively. Juvenile swimming crabs (initial weight 8.20 ± 0.01 g) were fed these experimental diets for 56 days. The findings indicated that the color of the live crab shells and the cooked crab shells gradually became red with the increase of dietary AST levels. Dietary 24.2 mg kg^−1^ astaxanthin significantly improved the growth performance of swimming crab. the lowest activities of glutathione (GSH), total antioxidant capacity (T-AOC), superoxide dismutase (SOD) and peroxidase (POD) were found in crabs fed without AST supplementation diet. Crabs fed diet without AST supplementation showed lower lipid content and the activity of fatty acid synthetase (FAS) in hepatopancreas than those fed diets with AST supplementation, however, lipid content in muscle and the activity of carnitine palmitoyl transferase (CPT) in hepatopancreas were not significantly affected by dietary AST levels. And it can be found in oil red O staining that dietary 24.2 and 45.8 mg kg^−1^ astaxanthin significantly promoted the lipid accumulation of hepatopancreas. Crabs fed diet with 195.0 mg kg^−1^ AST exhibited lower expression of *ampk*, *foxo*, *pi3k*, *akt* and *nadph* in hepatopancreas than those fed the other diets, however, the expression of genes related to antioxidant such as *cMn-sod*, *gsh-px*, *cat*, *trx* and *gst* in hepatopancreas significantly down-regulated with the increase of dietary AST levels. In conclusion, dietary 24.2 and 45.8 mg kg^−1^ astaxanthin significantly promoted the lipid accumulation of hepatopancreas and im-proved the antioxidant and immune capacity of hemolymph.

## 1. Introduction

Among invertebrates, crustaceans contain more carotenoids, and they can get this pigment by ingesting a variety of astaxanthin producing algae and microorganisms [1]. Astaxanthin is a naturally occurring fat-soluble red-orange oxidized carotenoid that was first isolated from the shells of shrimp and crabs in the 1930s and applied to aquaculture [2]. Crustaceans themselves cannot synthesize astaxanthin de novo [3], but they can obtain appropriate coloration by converting exogenous carotenoids in their diet to astaxanthin with synthetic canthaxanthin [4], or by directly store other carotenoids and astaxanthin in their diet. Therefore, the composition of feed has a great influence on the color of crustaceans, especially shells [5]. The structure of astaxanthin consists of a long non-polar conjugated polyene backbone connected to polar ionone rings located at each end which contains polar hydroxyl and carbonyl ionone rings, so it can exert its antioxidant effect by extinguishing singlet oxygen and eliminating free radicals [6,7].

A large number of studies have shown that astaxanthin (AST) exerts its antioxidant effect through a variety of mechanisms. These include regulating the *PI3K*/*Akt* pathway to directly clear reactive oxygen species (ROS) from the surface of cell membranes, and to protect mitochondrial functional integrity and REDOX balance (Nrf2)/antioxidant response elements (AREs) pathways by activating nuclear factor e2 associated Factor 2 (Nrf2)/antioxidant [8,9]. The *PI3K*/*Akt* signaling pathway influences multiple downstream pathways by directly regulating mitochondrial cascades and activating *NADPH*, or alternatively, by producing ROS as metabolic by-products, consequently leading to elevated ROS levels [10]. In crustaceans, several studies have demonstrated that the inclusion of a natural source of astaxanthin, incorporating *Haematococcus pluvialis* cell powder into the diet of Chinese mitten crab (*Eriocheir sinensis*) can significantly improve coloration, antioxidant capacity and proximate composition [11,12,13]. According to Niu et al. [9], dietary astaxanthin supplementation has been shown to improve the growth performance and increase resistance to pathogenic bacteria of Pacific white shrimp (*Litopenaeus vannamei*). Previous study reported that adding natural astaxanthin to the feed improved the digestive efficiency, antioxidant performance and immune response of juvenile red swamp crayfish (*Procambarus clarkii*), while also enhancing their resilience to the challenges of air exposure during transport [14]. Moreover, dietary 60 mg kg^−1^ microalgae astaxanthin supplementation could significantly improve the growth performance and survival of juvenile Chinese mitten crab, *Eriocheir sinensis*, as well as enhance antioxidant capacity, non-specific immunity, tissue astaxanthin content and resistance to ammonia nitrogen stress [15].

When astaxanthin was added into the diet, the symptoms of blood lymphocyte apoptosis and hepatopancreas structure damage caused by high pH stress were generally improved for swimming crab (*Portunus trituberculatus*) [16]. Dietary astaxanthin supplementation can reduce oxidative stress and increase the concentration of n-3 high unsaturated fatty acids (HU-FA) in the whole body, thereby improving the nutritional quality of products and promoting health [17]. Dietary astaxanthin supplementation can increase the activities of total antioxidant capacity (T-AOC), glutathione peroxidase (GPx) and acid phosphatase (ACP) in hemolymphatic, and increase the contents of total lipids and total carbohydrates in edible tissues of *P. tritumetatus* [18]. Meanwhile, dietary astaxanthin supplementation has the potential to affect glucose and lipid metabolism, adding astaxanthin to high-fat diet can significantly promote the growth, and reduce its liver index and abdominal fat percentage of largemouth bass (*Micropterus salmoides*) [19]. In the tiger puffer fish (*Takifugu rubripes*), dietary astaxanthin supplementation enhanced the activation of hepatic lipolysis and *β*-oxidation genes, while concurrently suppressing the activity of lipogenic-related genes [20]. However, there are few studies on astaxanthin in lipid metabolism in crustaceans, and it is unclear whether the above findings also exist.

The swimming crab (*Portunus trituberculatus*) occupies an important position among the marine crustaceans in China. Due to its delicious taste and culinary diversity, the species is highly sought after both for local consumption and as a valuable ingredient in the booming processing industry [21]. However, the area of scientific research into the nutritional quality of these crustaceans remains relatively unexplored. The aim of present study was to investigate the effects of different levels of astaxanthin on growth performance, antioxidant capacity and lipid metabolism of swimming crab.

## 2. Materials and Methods

### 2.1. Diets Preparation

**Figure 1:**
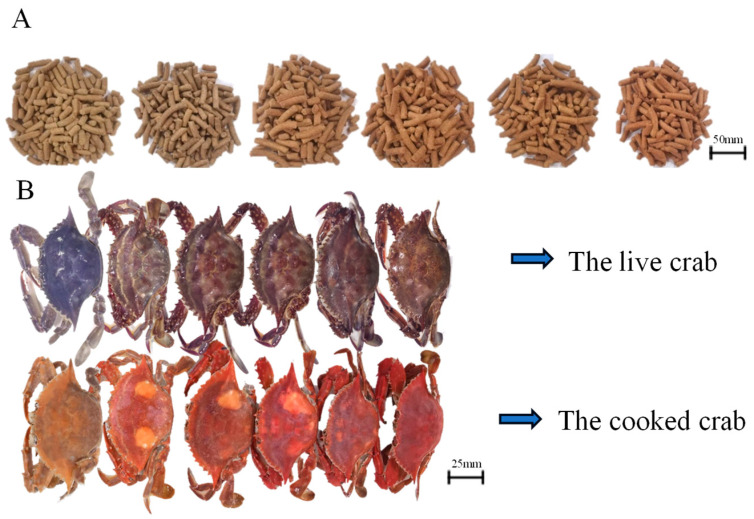
Effects of dietary astaxanthin level on carapace color of juvenile *Portunus trituberculatus*. The color of diet (**A**), from left to right with 0, 24.2, 45.8, 72.4, 94.2 and 195.0 mg/kg astaxanthin, the scale is 1:10; (**B**): from left to right, crabs were fed diets with 0, 24.2, 45.8, 72.4, 94.2 and 195.0 mg/kg astaxanthin, the scale is 1:5.

Six isonitrogenous and isolipidic experimental diets were developed to contain increasing astaxanthin levels of 0 mg/kg, 24.2 mg/kg, 45.8 mg/kg, 72.4 mg/kg, 94.2 mg/kg and 195 mg/kg (according to the measured value to marker as groups of 0, 24.2, 45.8, 72.4, 94.2 and 195.0), respectively. The astaxanthin (AST) utilized in this study was sourced from a powder containing 10% AST, obtained from a reputable commercial company (DSM, Shanghai, China). The main protein sources used in this study were Peruvian fish meal and soybean meal and fish oil and soybean lecithin were utilized as the main lipid sources, with wheat flour being employed as the exclusive source of carbohydrates (refer to Table 1). The premix is formulated according Xie et al. [22]. The dietary components were all finely pulverized and mixed evenly step by step ac-cording to dietary formula after passing 60-mesh sieve. Subsequently, the oil source was incorporated and thoroughly mixed. Then mixed in a Hobart-type mixer and cold-extruded pellets produced (F-26, Machine factory of South China University of Technology. Guangzhou, China) with pellet strands cut into uniform sizes (diameter 3 mm and 5 mm) (G-250, Machine factory of South China University of Technology). Pellets were steamed for 8 h at 45 °C, and then airdried to approximately 9% moisture, sealed in vacuum-packed bags and stored at −20 °C until used in the feeding trial (Luo et al.) [23]. The color of the feed gradually turned red as the amount of astaxanthin added increased, as shown in Figure 1A.

Juvenile crabs were acquired from the Xianxiang, Ningbo, Zhejiang Province and the culture was carried out in the crab apartment on the second floor of the pilot base of Meishan Campus of Ningbo University. Each experimental group was comprised of four replicates, each containing eight crabs. A total of 192 crabs, similar in size (8.20 ± 0.01 g/crab). The allocation of crabs into each cubicle was done randomly. Throughout the experimental period, crabs were provided with a daily ration equivalent to 6–8% of their body weight, which was fed the diet at 6.00 pm every day, cleaned up the leftover diet of the previous day at 8.00 am the next day. The number of dead and molting crabs and remaining feed was recorded and removed before feeding. The average water temperature maintained a steady level of 25.9 ± 0.3 °C, while the salinity remained consistent at 24.7 ± 0.2 g/L.

### 2.2. Sample Collection

Upon the conclusion of the 8-week feeding trial, the surviving crabs in each replicate were meticulously tallied and their weights recorded. The collected data was used to determine important factors including survival rate, final weight (FW), percent weight gain (PWG), specific growth rate (SGR), and feed efficiency (FE). For each trial, a group of six crabs was chosen for hemolymph sampling. Following an overnight incubation period, the coagulated portion was separated, and the remaining liquid was preserved at −80 °C for future examination. Meanwhile, hepatopancreas and intestine samples from six crabs in each replicate were quick-freeze in liquid nitrogen, and then stored at −80 °C. And the remaining crabs in each group were boiled at 100 °C for 5 min to observe the color change show in Figure 1B.

### 2.3. Fatty Acids Analysis in Hepatopancreas 

The method described by Jin et al. [24] was employed to analyze the fatty acid composition of the hepatopancreas. Samples that underwent freeze-drying were placed in a 12 mL volumetric glass screw tube with a lid and a Teflon gasket. Following this, methanol containing 1 N potassium hydroxide was introduced in a volume of 3 mL and the blend was subjected to incubation at a temperature of 72 °C in a water bath for a duration of 20 min. The cooling was applied, followed by the addition of 3 mL of methanol containing HCl at a concentration of 2 N, the blend underwent the process of being warmed up again at 72 °C for an additional 20 min. Previous tests validated the effectiveness of these esterification techniques for all fatty acids. Following the addition of 1 mL of hexane, the mixture was vigorously shaken for 1 min and then left to separate into two layers. Subsequently, the fatty acid methyl esters were isolated and quantified using gas chromatography-mass spectrometry GC-MS (Agilent Technologies 7890B-5977A instrument, Santa Clara, CA, USA).

### 2.4. Antioxidant and Non-Specific Immune-Related Enzyme Activities

Weigh approximately 0.5 g of hepatopancreas samples, transfer them to a centrifuge tube, and then add pre-cooled normal saline (PH7.0–7.5) in a 1:9 ratio. Homogenize the mixture using a tissue homogenizer. Centrifuge the homogenized solution at 8000 rpm at 4 °C for 15 min, and collect the supernatant as crude enzyme liquid for further analysis. Extract the hemolymph, refrigerate it for 12 h until it reaches a jelly-like consistency. After thoroughly mashing the hemolymph with a needle, centrifuge it at 8000 rpm at 4 °C for 15 min. Collect the upper layer of hemolymph and store it in a −80 °C refrigerator for subsequent analysis. The primary methods for detecting antioxidant enzymes include measuring superoxide dismutase (SOD) activity (reaction temperature: 25 °C) using the WST-1 method, determining the concentrations of glutathione (GSH) (reaction temperature: 25 ℃) and nitric oxide (NO) (reaction temperature: 37 °C), as well as acid phosphatase (ACP) and alkaline phosphatase (AKP) enzyme activities (reaction temperature: 37 °C) through the microplate method. Additionally, glutathione peroxidase (GPX) (reaction temperature: 25 °C), peroxidase (POD) (reaction temperature: 37 °C), and nitric oxide synthase (NOS) activities (reaction temperature: 37 °C) are assessed using the colorimetric method, while total antioxidative capacity (T-AOC) activity (reaction temperature: 37 °C) is determined using the ABTS method. The kits utilized for these tests were sourced from Nanjing Jiancheng Bioengineering Institute in China, with test procedures conducted in accordance with the kit instructions. Catalase (CAT) levels (reaction temperature: 37 °C) in both hemolymph and hepatopancreas were evaluated using a commercial kit from Qiaodu Biotechnology (Shanghai, China).

### 2.5. Lipid Metabolism-Related Enzyme Activities and Crude Lipid Content of Hepatopancreatic and Muscle

Hepatopancreatic lipid metabolism-related enzyme activities included fatty acid synthase (FAS) (reaction temperature: 37 °C) and carnitine palmitoyl transferase (CPT) (reaction temperature: 37 °C) were assayed by the commercial kits (Shanghai Qiaodu Biotechnology, China). The hepatopancreas supernatant was mixed with the reactants and recorded the absorbance corrected at 450 nm. The Spectramax m2 spectrophotometer was used enzyme-Linked Immunosorbent Assay’s (ELISA) technical readings of optical density. The crude lipid content of hepatopancreatic and muscle samples were analyzed using the methods outlined of the Association of Official Analytical Chemists (AOAC) [25] by using the Soxhlet method (SX360, OPSIS).

### 2.6. Tissue Section and Observation

Assessment was conducted on hepatopancreas samples taken from three individual crabs representing each dietary treatment, with one sample from each dietary replicate. Five measurements were taken for each hepatopancreas sample. The hepatopancreas samples underwent paraffin sectioning and histological analysis at Haoke Biological Co., Ltd. in Hangzhou, China). Following fixation in 4% paraformaldehyde for a minimum of 48 h, the hepatopancreas samples were dehydrated, embedded in paraffin and then cut into 4 µm sections. The sections embedded in paraffin were then deparaffinized in xylene, gradually rehydrated with ethanol, and stained with hematoxylin-eosin (HE) dye (Beyotime, Shanghai, China). After a series of ethanol dehydration steps, the sections were sealed with neutral resin. Histology images were captured using a Pannoramic MIDI pathology section scanner (Tangier, Jinan, China).

Following fixation in 4% paraformaldehyde for a minimum of 48 h, the hepatopancreas tissues were dehydrated, immersed in OCT medium, and then cut into slices with a thickness of 8−10 µm. These sections were then subjected to staining with Oil Red O for 15 min, ensuring they were kept away from light, and sealed with glycerin gelatin. The histology images were acquired using a Panoramic MIDI pathology section scanner.

### 2.7. Quantitative RT-PCR 

The quantitative RT-PCR method described by Yang et al. [26]. In brief, total RNA was extracted from pooled hepatopancreas and intestinal samples using Trizol reagent (Vazyme Biotechnology Co., Ltd. Nanjing, China) according to the manufacturer’s protocol. The quality and concentration of the extracted RNA were assessed via 1.2% agarose gel electrophoresis and ultra-micro spectrophotometer (Nanodrop 2000, Thermo Fisher Scientific, Shanghai, China USA), respectively. Subsequently, the RNA samples were reverse transcribed into cDNA at a concentration of 1000 ng ml^−1^ using the HiScript II-RT Reagent Kit (Vazyme Biotech Co., Ltd., Nanjing, China), following the manufacturer’s instructions. The resulting cDNA was then diluted fourfold with DEPC water and stored at −80 °C for subsequent use. Specific primers were designed based on the corresponding cDNA sequences obtained from the National Center for Biotechnology Information (NCBI) database using Primer Premier 5.0 software (Supplementary Table S1). Alignment curves were established from gradient dilutions of cDNA samples at six concentrations. The amplification efficiency was calculated as E = 10 (−1/Slope) − 1 and ranged from 80% to 110% for all genes. The absolute values of ΔCT (target gene—internal reference gene, *β*-actin) were consistently below 0.100, indicating comparable amplification efficiencies between the target gene and the internal reference gene. Reverse transcription quantitative polymerase chain reaction (RT-qPCR) was conducted on a quantitative thermocycler (LightCycler 96, Roche, Zurich, Switzerland) using 10 μL reaction mixtures comprising 1 μL diluted cDNA, 0.5 μL of each primer, 5 μL 2× concentrated SYBR Green I Master (Roche, Switzerland), and 3 μL DEPC-treated water. The qPCR protocol began with a pre-warming step at 95 °C for 2 min, followed by 45 cycles of amplification at 95 °C for 10 s, 58 °C for 10 s, and 72 °C for 20 s. Relative expression levels of the target genes were determined using the 2^−ΔΔCt^ method by Livak and Schmittgen [27]. The *β*-actin gene was chosen as the housekeeping gene due to its stability and the 0 mg kg^−1^ astaxanthin diet was used as the control (reference) group.

### 2.8. Statistical Analysis

The parameters were calculated as follows:

Percent weight gain (PWG, %) = 100 × (average final body weight of crab − average initial body weight of crab)/average initial body weight of crab

Specific growth rate (SGR, % day^−1^) = 100 × (Ln average final body weight of crab − Ln average initial body weight of crab)/feeding days

Feed efficiency (FE) = (final total weights + dead crab total weights − initial total weights)/total feed intake

Survival (%) = 100 × final number of crabs/initial number of crabs

Molting rate (MR) = 2 × molting number/(initial number of crabs + final number of crabs)

Total feed intake was the ration given minus the residual (uneaten) feed. The results were presented as means ± S.E.M. (n = 3). One-way analysis of variance (ANOVA) was used to test for the significance of differences between treatments in individual parameters. SPSS 20.0 software (SPSS Inc., Chicago, IL, USA) was used for statistical analysis of the data.

## 3. Results

### 3.1. Color Parameters

The effect of dietary astaxanthin levels on carapace color was shown in Figure 1. The carapace color of live crabs and cooked crabs significantly became red with dietary astaxanthin levels increasing from 0.0 to 195.0 mg kg^−1^. 

### 3.2. Growth Performance and Survival

**Figure 2:**
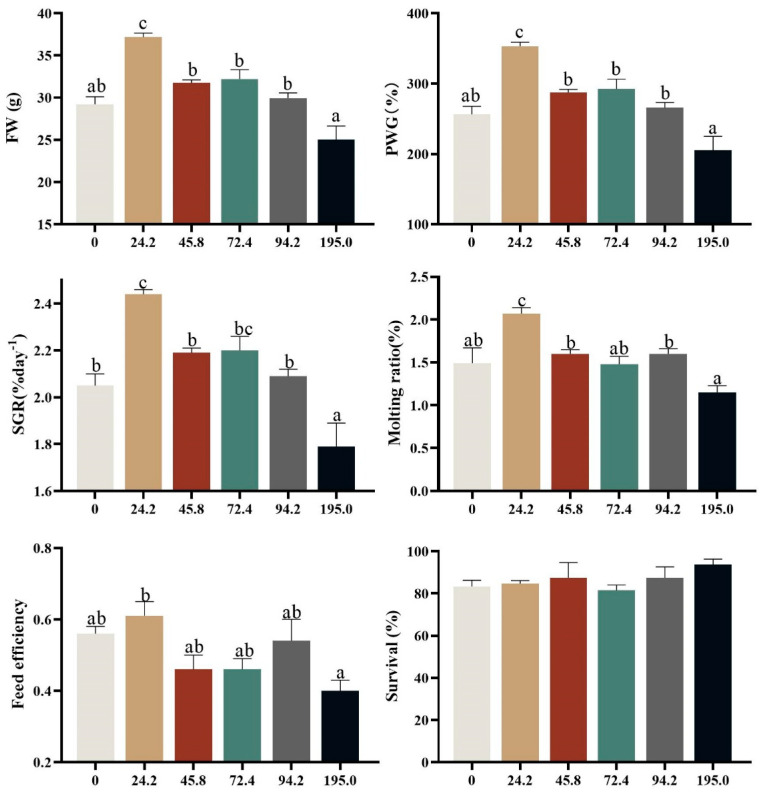
Growth performance, survival and molting rate of *Portunus trituberculatus* fed different AST levels. Data are presented as means ± SEM of three replicates. Mean values in the same row with different superscript letters are significantly different (*p* < 0.05). FW, final weight; PWG, percent weight gain; SGR, specific growth rate; MR, Molting ratio.

Effects of dietary astaxanthin levels on growth, survival and feed utilization were presented in Figure 2. Crabs fed diet with 24.2 mg kg^−1^ astaxanthin exhibited higher percent weight gain (PWG), specific growth rate (SGR), molting ratio (MR) and feed efficiency (FE) than those fed the other diets, and the lowest PWG, SGR, MR and FE were observed at crabs fed diet without astaxanthin supplementation (*p* < 0.05). There was no significant difference on survival among all treatments (*p* > 0.05).

### 3.3. Composition of Fatty Acids in the Hepatopancreas

The fatty acid composition of *P. trituberculatus* fed diets with different astaxanthin were exhibited in Table 2. Crabs fed diet with 195.0 mg kg^−1^ astaxanthin showed lower total saturated fatty acids (SFAs), monounsaturated fatty acids (MUFAs), n-6 and n-3 poly unsaturated fatty acids (n-6 PUFA and n-3 PUFA) in hepatopancreas than those fed the other diets (*p* < 0.05). The highest DHA concentration in hepatopancreas was occurred at crabs fed diets with 72.4 and 94.2 mg kg^−1^ astaxanthin (*p* < 0.05). 

### 3.4. Enzymes Activities of Non-Specific Immunity and Antioxidant in Hemolymph

**Figure 3:**
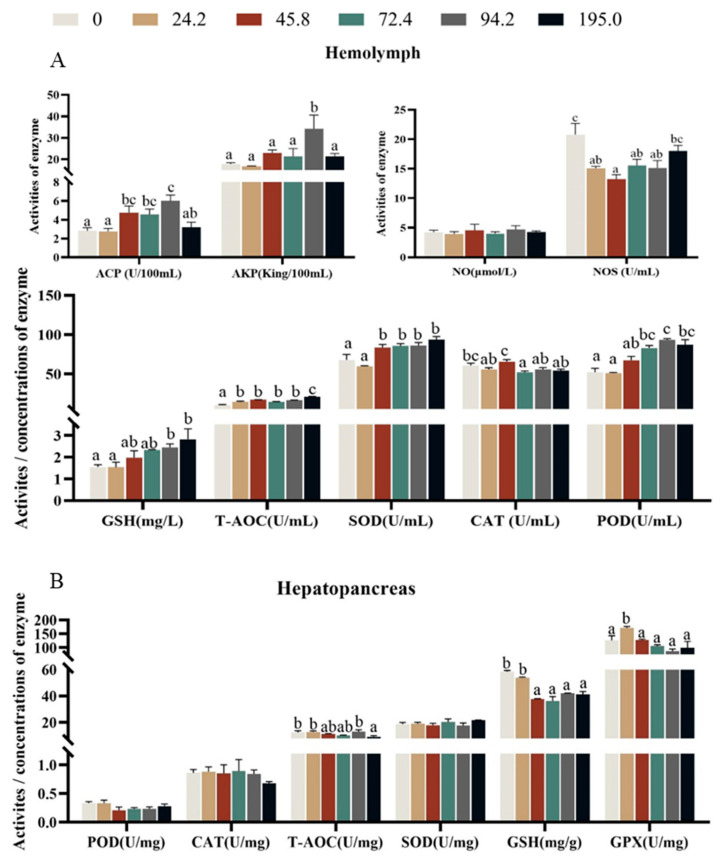
Effects of dietary astaxanthin levels on antioxidant and immune-related enzyme activity of juvenile *Portunus trituberculatus* fed the experimental diets for 8 weeks in hemolymph (**A**) and hepatopancreas (**B**). Data are presented as means ± SEM of three replicates. Mean values in the same row with different superscript letters are significantly different (*p* < 0.05). ACP, acid phosphatase; AKP, alkaline phosphatase; NO, nitric oxide; NOS, nitric oxide synthase; GSH, glutathione; SOD, superoxide dismutase; T-AOC, total antioxidative capacity; POD, peroxidase; GPX, glutathione peroxidase; CAT, catalase.

Crabs fed diet with 94.2 mg kg^−1^ astaxanthin had higher activities of acid phosphatase (ACP) and alkaline phosphatase (AKP) in hemolymph than those fed the other diets (Figure 3A, *p* < 0.05), however, the activity of nitric oxide synthase (NOS) significantly decreased with dietary astaxanthin levels increasing from 0.0 to 45.8 mg kg^−1^, and then significantly increased with the further increase of dietary astaxanthin (Figure 3A, *p* < 0.05). However, nitric oxide (NO) in hemolymph was not significantly influenced by dietary astaxanthin levels. The activities of GSH and POD in hemolymph significantly increased with the increase of dietary astaxanthin levels (*p* < 0.05). Crabs fed diet without astaxanthin supplementation exhibited lower activities of T-AOC and SOD in hemolymph than those fed the other diets (Figure 3A, *p* < 0.05).

The activities of T-AOC, GSH and GPX in hepatopancreas significantly decreased with increase of dietary astaxanthin levels (Figure 3B, *p* < 0.05), however, the activities of POD, CAT and SOD in hepatopancreas were not significantly influenced by dietary astaxanthin levels (*p* > 0.05).

### 3.5. The Activities of Enzymes Related to Lipid Metabolism in Hepatopancreas and Oil Red Staining

**Figure 4:**
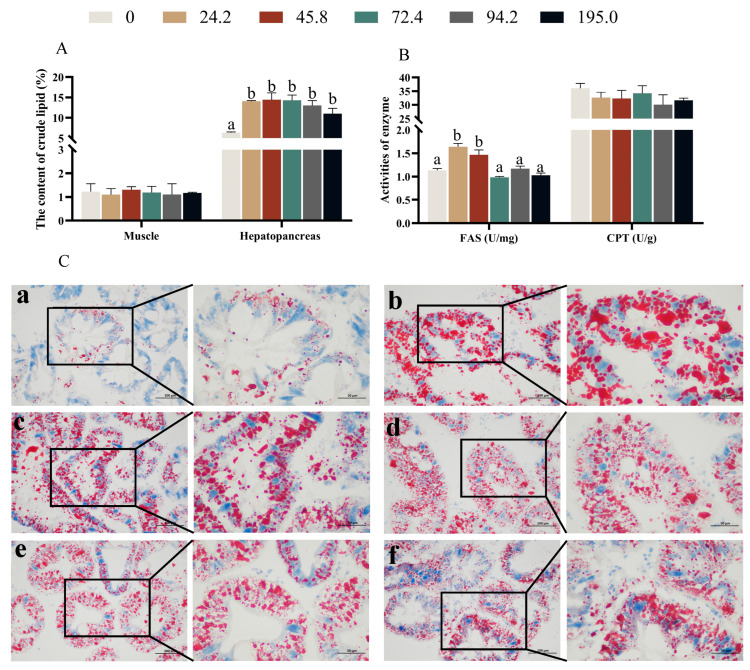
Effects of dietary astaxanthin levels on lipid metabolism of juvenile *Portunus trituberculatus* fed the experimental diets for 8 weeks. (**A**) crude lipid content of hepatopancreatic and muscle; (**B**) Lipid metabolism-related enzyme activities in hepatopancreas. Data are presented as means ± SEM of three replicates. Mean values in the same row with different superscript letters are significantly different (*p* < 0.05). FAS, fatty acid synthase; CPT, carnitine palmitoyl transferase; (**C**) represent sections stained with oil red O staining photographed under 200× and 400× light microscope (scale bar, 100 μm or 50 μm respectively) in hepatopancreas, effects of dietary astaxanthin at (**a**) 0, (**b**) 24.2, (**c**) 45.8, (**d**) 72.4, (**e**) 94.2 and (**f**) 195.0 mg/kg respectively.

Crabs fed diet without astaxanthin supplementation showed lower lipid content in hepatopancreas than those fed the other diets (Figure 4A, *p* < 0.05), however, lipid content in muscle was not significantly affected by dietary astaxanthin levels. The highest activity of fatty acid synthase (FAS) in hepatopancreas were observed at crabs fed diets with 24.2 and 45.8 mg kg^−1^ astaxanthin (Figure 4B, *p* < 0.05), moreover, there was no significant difference on the activity of carnitine palmitoyl transferase (CPT) in hepatopancreas. In the oil red O staining, it was found that the group with added astaxanthin showed a significant increase in the number of lipid droplets in hepatopancreas (Figure 4C). 

### 3.6. Histological Structure in Hepatopancreas

**Figure 5:**
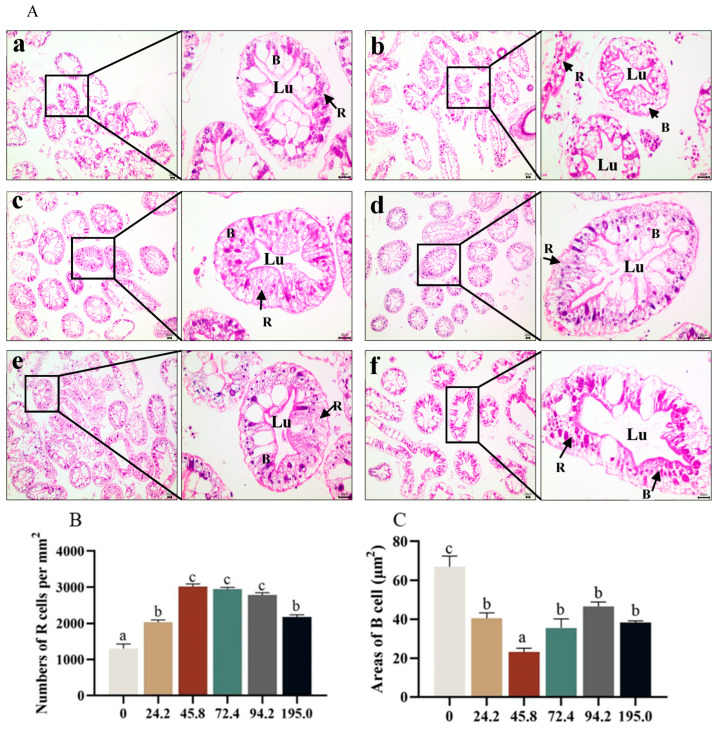
Effects of dietary astaxanthin on hepatopancreas histological structure of *Portunus trituberculatus* fed the experimental diets for 8 weeks (**A**), effects of dietary astaxanthin at (**a**) 0, (**b**) 24.2, (**c**) 45.8, (**d**) 72.4, (**e**) 94.2 and (**f**) 195.0 mg/kg respectively. Scale bar, 20 μm; 100× and 400× magnification). Data on R cells are provided in panels (**B**) numbers, (**C**) areas of B cell (n = 40), R (restzellen) cell; B, B (blasenzellen) cell; Lu, lumen structure. The arrows are showing where the R (restzellen) cell and B (blasenzellen) cells are located. Data are presented as means ± SEM of three replicates. Mean values in the same row with different superscript letters are significantly different (*p* < 0.05).

The quantitative analysis of hepatopancreatic R cell characteristics revealed a significant increase in the number of R cells with the dietary astaxanthin level increasing from 0 to 45.8 mg kg^−1^, however, the number of R cells significantly decreased with dietary astaxanthin increasing from 94.2 to 195.0 mg kg^−1^ (Figure 5A,B, *p* < 0.05). The area of B cells in the hepatopancreas decreased significantly with dietary astaxanthin levels increasing from 0.0 to 45.8 mg kg^−1^, and then increased significantly with further increase of dietary astaxanthin levels (Figure 5C, *p* < 0.05).

### 3.7. The Expression of Genes Related to Immune-Related in Intestinal

**Figure 6:**
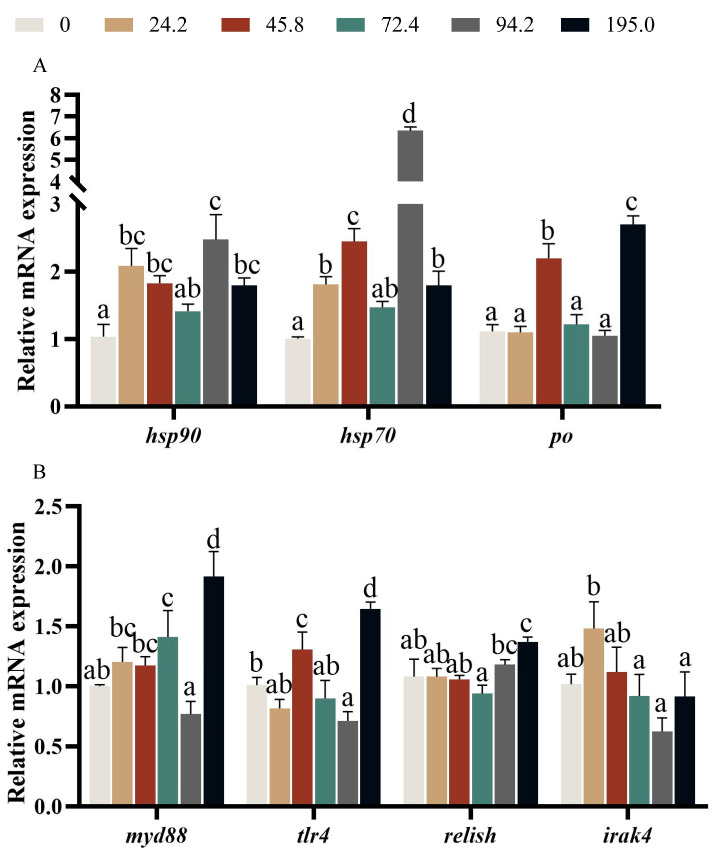
Relative expression of genes related to non-specific immunity in intestine (**A**,**B**) of *Portunus trituberculatus*. Data are presented as means ± SEM of three replicates. Mean values in the same row with different superscript letters are significantly different (*p* < 0.05). *irak4*, *IL-1* receptor–associated kinase 4; *myd88*, myeloid differentiation factor 88; *hsp70*, heat shock protein 70; *hsp90*, heat shock protein 90; *tlr4*, toll like receptor 4; *relish*, target of rapamycin; *po*, phenol oxidase.

Crabs fed diet with 94.8 mg kg^−1^ astaxanthin exhibited higher expression of *hsp70* and *hsp90* in intestinal than those fed the other diets, however, the highest expression of *po* was observed at crabs fed diet with 195.0 mg kg^−1^ astaxanthin (Figure 6A, *p* < 0.05).

The highest expressions of *myd88*, *tlr4* and *relish* were occurred at crabs fed diet with 195.0 mg kg^−1^ astaxanthin (Figure 6B, *p* < 0.05). Crabs fed diet with 24.2 mg kg^−1^ astaxanthin exhibited higher expression of *irak4* than those fed diets with 72.4, 94.2 and 195.0 mg kg^−1^ astaxanthin (Figure 6B, *p* < 0.05).

### 3.8. Expression of Genes Related to Antioxidant and Fatty Acid Metabolism in Hepatopancreas

**Figure 7:**
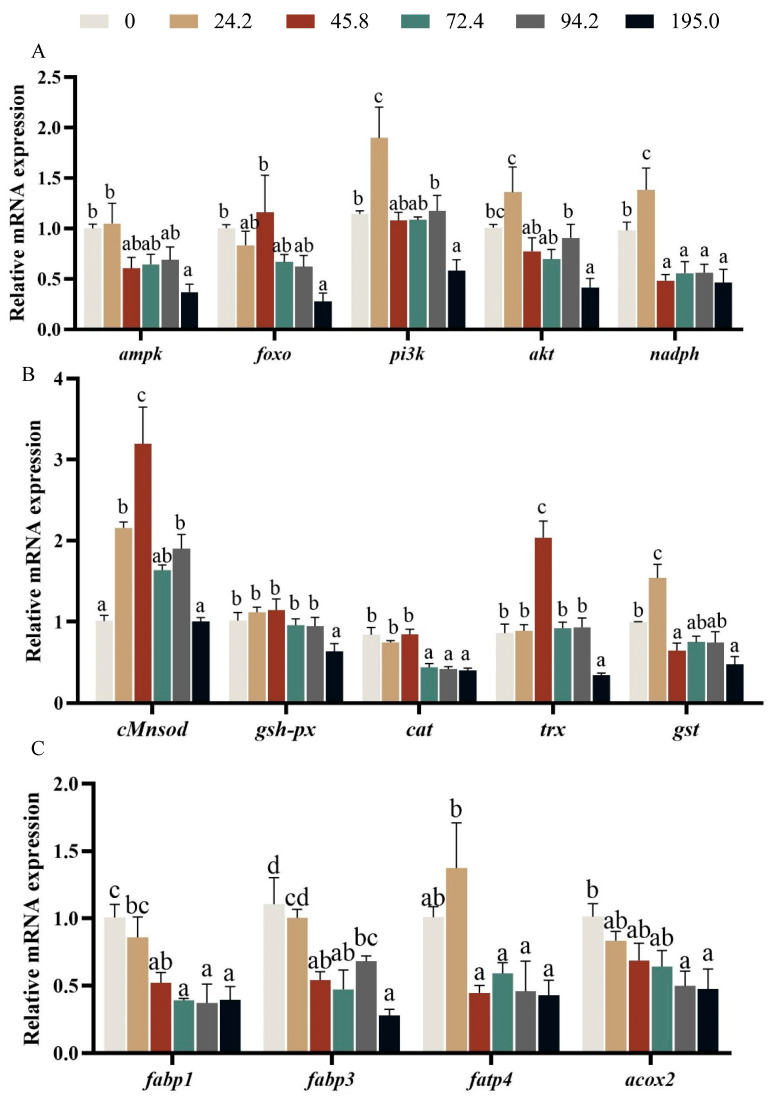
Effects of dietary astaxanthin level on the expression levels of genes related to antioxidant (**A**,**B**) and fatty acid transport metabolism (**C**) in hepatopancreas of *Portunus trituberculatus* fed the experimental diets for 8 weeks. Data are presented as means ± SEM of three replicates. Mean values in the same row with different superscript letters are significantly different (*p* < 0.05). *cMnsod*, superoxide dismutase; *cat*, catalase; *gsh-px*, glutathione peroxidase; *ampk*, Adenosine 5′-monophosphate (AMP)-activated protein kinase α2; *foxo*, forkhead box protein O1; *pi3k*, Phosphoinositide 3-kinase; *akt*, protein kinase B; *nadph*, nicotinamide adenine dinucleotide phosphate; *gst*, Glutathione S-transferase; *fabp1*, fatty acid binding protein 1; *fabp3*, fatty acid binding protein 3; *fatp4*, fatty acid transport protein 4; *acox2*, acyl-coenzyme A oxidase 2.

Crabs fed diet with 24.2 mg kg^−1^ astaxanthin exhibited higher expression of *pi3k*, *akt* and *nadph* than those fed the other diets, and the lowest expression of *ampk*, *foxo*, *pi3k*, *akt* and *nadph* were found at crabs fed diet with 195.0 mg kg^−1^ astaxanthin (Figure 7A, *p* < 0.05).

Crabs fed diet with 45.80 mg kg^−1^ astaxanthin showed significantly higher expression of *cMnsod* and *trx* in hepatopancreas than those fed the other diets (Figure 7B, *p* < 0.05). The expression of *gsh-px*, *cat* and *gst* significantly down-regulated with the increase of dietary astaxanthin levels, and crabs fed diet with 195.0 mg kg^−1^ astaxanthin had significantly lower expression of *cat* and *gsh-px* than those fed the other diets (Figure 7B, *p* < 0.05). The expression of genes related to transport metabolism such as *fabp1*, *fabp3*, *fabp4* and *acox2* significantly down-regulated with the increase of dietary astaxanthin levels, and the highest expression of *fabp1*, *fabp3* and *acox2* were observed at crabs fed diet without astaxanthin supplementation (Figure 7C, *p* < 0.05).

## 4. Discussion

Astaxanthin, as an important nutritional supplement, has been extensively studied for its beneficial effects on promoting the growth and survival of aquatic organisms. Over time, studies on astaxanthin have yielded a variety of findings, most of which have focused on crustaceans [15,28]. More studies have shown that adding astaxanthin to feed can improve the growth and survival of fish and crustaceans [29]. However, some studies have failed to observe any significant enhancement in growth and survival of various aquatic organisms after adding astaxanthin to diets [30,31]. The results of present study indicated that dietary 24.20 mg kg^−1^ astaxanthin could significantly improve the growth performance of swimming crab *P. trituberculatus*. Similarly, adding 25 to 100 mg kg^−1^ astaxanthin to the diet can significantly improve the weight gain of *Penaeus monodon*, but has no significant effect on the survival [32]. Adding 380 mg kg^−1^ astaxanthin diet can significantly improve the survival of red king crab *Paralithodes camtschaticus* [33]. This compelling evidence consistently highlights the importance of dietary astaxanthin as a key nutritional additive to promote the growth and survival of aquatic organisms.

Previous studies demonstrated that dietary astaxanthin supplementation could improve the coloration of crabs. In Chinese mitten crab (*Eriocheir sinensis*), a diet rich in astaxanthin caused their shells to take on a deeper, more vivid red color, and likewise, red king crabs fed a diet rich in astaxanthin showed a more intense red color [11,12]. In this study, it was also found that dietary astaxanthin significantly improved the shell color of living crabs. After cooking at 100 °C for 5 min, with the increase of dietary astaxanthin, the shell redness of *P. trituberculatus* was significantly deepened. In contrast, adding carotenoids or astaxanthin to the diet had no effect on skin brightness (L*) in Australian snapper (*Pagrus auratus*) and large yellow croceus (*Larimichthys croceus*) [34,35]. The change of body color is affected by many factors and is a responsible process. The effect of dietary astaxanthin supplementation on the coloration of shell, hepatopancreas and gonads of crustaceans deserves further study.

Astaxanthin plays a crucial role in the cellular metabolism of marine organisms, and its antioxidant properties are closely related to the improvement of animal survival. The maintenance of cell homeostasis is largely dependent on the regulation of autophagy, promoting optimal cell function and enhancing physiological antioxidant capacity in response to various stress conditions [29]. Astaxanthin is known for its powerful antioxidant capacity due to its unique molecular structure filled with conjugated double bond chains modified by unsaturated carbonyl and hydroxyl groups. This ingenious arrangement gives it the ability to provide unpaired electrons that diligently neutralize harmful free radicals that attack the body [36,37]. The antioxidant defense system of crustaceans is composed of two major systems: enzymatic and non-enzymatic, the enzymatic system includes antioxidant enzymes such as SOD, CAT and GPX, while the non-enzymatic system includes vitamins, carotenoids, amino acids, proteins and metals, which can protect the body from free radicals under the synergistic interaction [38]. Some studies have shown that excessive addition of antioxidants to feed can have toxic effects on long-term aquaculture animals [39,40]. The main reason may be that the body cannot adapt to high levels of antioxidants, leading to a stress response, increasing the production of ROS in the body, indirectly damaging the cell membrane and organelles (endoplasmic reticulum, mitochondria), thereby reducing the body’s immunity and growth ability [41]. Research on Chinese mitten crabs has shown that excessive supplementation of vitamin A can have a negative impact on antioxidant enzyme activity to a certain extent [42]. In this study, with the increase of dietary astaxanthin levels, the activities of most antioxidant enzymes in hemolymph significantly increased, and the activities of GSH, T-AOC and SOD reached the maximum value in crabs fed diet with 195.0 mg kg^−1^ astaxanthin. The results of present study were consistent with those of *Litopenaeus vannamei* [38]. The activities of T-AOC, GPX and GSH in hepatopancreas significantly decreased with dietary astaxanthin levels increasing from 24.2 to 45.8 mg kg^−1^ astaxanthin. The reason may be that after ingestion of astaxanthin, crabs participate in the antioxidant process of the body as a non-enzymatic component, clearing excessive oxygen free radicals in the hemolymph, reducing the reaction substrate of antioxidant enzymes, and ultimately leading to a decline in its activity in hepatopancreas [43]. The potential factors contributing to the diminution of its antioxidant capacity are twofold. Firstly, as dietary astaxanthin supplementation escalates, there is a discernible augmentation in its deposition within the hepatopancreas, which, in turn, leads to a more efficient sequestration of astaxanthin. Secondly, the excessive presence of astaxanthin may engender its oxidative degradation, thereby inadvertently attracting a greater influx of free radicals.

The immune system of crustaceans often faces a variety of challenges from the surrounding environment, including protozoa, bacteria, viruses, and various kinds of stress [15,44]. Crustaceans tend to accumulate large amounts of carotenoids, especially astaxanthin, in their tissues. This accumulation is thought to regulate immunopathology and findings’ immune function, thereby improving the ability to adapt and accept environmental stress [45]. AKP and ACP are key phosphatases that control many immune functions in all organisms, and their activity is a barometer of the immune status of crustaceans [46,47]. In the present study, it was observed that the ACP and AKP activities of hemolymph increased significantly with the increase of dietary astaxanthin. The above important evidence showed that adding astaxanthin to the diet has a positive effect on the immune response of crabs. This finding was consistent with previous study on adult swimming crab [48], juvenile yellow perch (*Perca flavescens*) [49], and juvenile red swamp crayfish (*Procambarus clarkii*) [50]. Nitric oxide (NO), which is considered as a novel biological messenger and effector and immune regulation molecules, extensively exists in many organs and tissues of the organism and involves in many physiological processes, especially in immune response [51]. In organisms, NO was produced from L-arginine in the presence of nitric oxide synthase [52]. As NOS activity is closely related to pathogen infection and environmental stress, it has been used as an indicator to reflect the healthy condition and immunity of crustacean [53]. With the increase of dietary astaxanthin content, the activity of nitric oxide synthase (NOS) firstly decreased and then increased. This may be because the swimming crab ingests astaxanthin, which increases the content of astaxanthin in the hemolymph, thereby enhancing the body’s immune capacity and replacing some of the functions of NOS. Precisely measuring the expression levels of immune-related genes allows us to assess how specific biological triggers promote immune response and health in aquatic organisms. However, there is little information on the actual role of astaxanthin in regulating the expression of immune-related genes in crustaceans [29]. The primary defense mechanism of animals is the innate immune system, which is activated by toll-like receptors (TLRs) that recognize conserved patterns of pathogenic microorganisms [54]. It is worth noting that MyD88 and TRF-dependent pathways are two widely studied TLR signal transduction methods [55,56]. MyD88 and *irak4* are key interacting components that initiate downstream gene expression in the MyD88-dependent pathway [57,58], while IRF3 and IRF7 are significant transcription factors involved in the TRIF-dependent pathway [59]. In the present study, the expression of *myd88* in the intestine was notably up-regulated at the crabs fed diets with 72.4 and 195.0 mg kg^−1^ astaxanthin. The crabs fed diet with 24.2 mg kg^−1^ astaxanthin exhibited higher expression level of *irak4* in the intestine than those fed diets with 72.4, 94.2 and 195.0 mg kg^−1^ astaxanthin.

Nuclear factor-erythroid 2 p45-related factor 2 (Nrf2) is a fine transcription factor that coordinates the complex balance of cellular redox states by simultaneously controlling key components in the endogenous antioxidant enzyme system, attracting more free radical inflow [60]. Previous studies demonstrated the indispensable role of Nrf2 in upholding redox homeostasis in organisms, primarily through augmenting the abundance of nicotinamide adenine dinucleotide phosphate (NADPH) to instigate the activation of thioredoxin (Trx)-dependent antioxidant systems [61]. Notably, thioredoxin (Trx) assumes a paramount position among the antioxidant proteins, endowed with remarkable oxidoreductase activities [62,63]. More recently, further studies have shown that adding astaxanthin to the diet enhances mRNA expression of genes related to antioxidant (*cat*, *cMnsod*, and *gpx*) in the hepatopancreas [64]. The finding suggested that a decrease in antioxidant capacity may lead to lipid peroxidation. In this study, the expression of sod and *trx* in crabs fed diet with 45.8 mg kg^−1^ astaxanthin were significantly up-regulated, while the expressions of *gsh-px*, *cat* and *gst* were significantly inhibited. The expression of *nadph* in crabs fed diet with 24.2 mg kg^−1^ astaxanthin was significantly up-regulated. These findings clearly showed that dietary astaxanthin supplementation effectively enhances the ability of juvenile crabs to resist oxidative stress and strengthens their immune systems. However, it is necessary to further explore the underlying mechanism.

The hepatopancreas, an integral organ for lipid metabolism, nutrient homeostasis, and energy reservoir, serves as a valuable indicator to discern alterations in dietary nutrition [65]. Hence, a greater manifestation of hepatopancreatic impairment becomes discernible in the histopathological sections of the hepatopancreas exhibited H.E. staining. The present study indicated that the number of hepatopancreas R cells was significantly increased in crabs fed diet with 45.8 mg kg^−1^ astaxanthin, while the hepatopancreas lumen was significantly deformed in crabs fed diet with 195.0 mg kg^−1^ astaxanthin. Crabs fed diet without astaxanthin dramatically displayed a lower crude lipid content in hepatopancreas than those fed the other diets. This finding was also supported by hepatopancreas oil red O staining, which found that lipid accumulation in hepatopancreas was significantly enhanced by dietary astaxanthin supplementation. In addition, the activity of enzymes associated with lipid synthesis in hepatopancreas, especially fatty acid synthetase (FAS), was significantly enhanced by dietary supplementation with 24.2 and 45.8 mg kg^−1^ astaxanthin. One possible explanation for the observed increase in crude lipid content may be related to the optimal concentration of astaxanthin in the tissue. Reaching an ideal concentration of astaxanthin may lead to a reduction in energy expenditure and an increase in lipid storage in the hepatopancreas, the vital organ responsible for absorbing and storing consumed lipids [66]. Wang et al. [67] also reported higher crude lipid content in hepatopancreas of Chinese mitten crabs fed diet with 68 mg kg^−1^ astaxanthin compared to the control diet. Conversely, Han et al. [31] noted a reduction in crude lipid content in swimming crabs following the consumption of astaxanthin-enriched diets. Due to limited information, the exact reasons for these different results remain elusive. However, an in-depth study of the metabolic and physiological characteristics of these carotenoids in specific species is essential.

To investigate this phenomenon, fatty acid content in hepatopancreas was analyzed, and most fatty acids in crab fed diet with 195.0 mg kg^−1^ astaxanthin were significantly lower than those fed the other diets. The lowest concentration of docosahexaenoic acid (DHA), omega-3 polyunsaturated fatty acids (n-3 PUFA), and omega-6 polyunsaturated fatty acids (n-6 PUFA) in hepatopancreas were observed at crabs fed diet with 195.0 mg kg^−1^ astaxanthin. Lipid profiles in tissues reflect the complex interplay between dietary fatty acid composition and their subsequent deposition in the body [68,69]. As the principal reservoir for lipid storage, the hepatopancreas serves as a faithful sentinel, faithfully capturing the nuances of dietary fatty acid deposition [70]. Furthermore, FABP3, a lipid chaperone par excellence, assumes pivotal responsibilities in orchestrating the intricate symphony of lipid metabolism [71]. In the present study, crabs fed diet with 195.0 mg kg^−1^ astaxanthin showed the lowest expression of genes related to fatty acid transport such as *fabp1*, *fabp3*, *fatp4* and *acox2* among all treatments. The results suggested that the excessive intake of astaxanthin can lead to the disturbance of hepatopancreas lipid metabolism and the decrease of overall energy metabolism. It is evidenced that crabs fed diet with 195.0 mg kg^−1^ astaxanthin exhibited lower expression of *ampk*, *foxo*, *pi3k* and *akt* than those fed the other diets.

## 5. Conclusions

In conclusion, dietary 24.2 mg kg^−1^ astaxanthin significantly increased the weight gain rate, specific growth rate and molt rate of swimming crab. Meanwhile, dietary 24.2 and 45.8 mg kg^−1^ astaxanthin significantly promoted the lipid accumulation of hepatopancreas and improved the antioxidant and immune capacity of hemolymph.

## Figures and Tables

**Table 1:** Ingredients and proximate composition of the experimental diets (dry basis, %).

Ingredients (Dry Weight, %)	Dietary Astaxanthin Levels (mg/kg)					
	0.0	24.2	45.8	72.4	94.2	195.0
Peru fish meal	30.00	30.00	30.00	30.00	30.00	30.00
Soybean meal	22.00	22.00	22.00	22.00	22.00	22.00
Soybean protein concentrate	6.00	6.00	6.00	6.00	6.00	6.00
Poultry by-product meal	5.00	5.00	5.00	5.00	5.00	5.00
Yeast extract	3.00	3.00	3.00	3.00	3.00	3.00
Wheat flour	22.00	22.00	22.00	22.00	22.00	22.00
Fish oil	3.00	3.00	3.00	3.00	3.00	3.00
Soybean lecithin	3.00	3.00	3.00	3.00	3.00	3.00
Cholesterol	0.50	0.50	0.50	0.50	0.50	0.50
Vitamin premix ^1^	0.50	0.50	0.50	0.50	0.50	0.50
Mineral premix ^2^	1.00	1.00	1.00	1.00	1.00	1.00
Ca(H_2_PO_4_) ^2^	1.00	1.00	1.00	1.00	1.00	1.00
Choline chloride	0.30	0.30	0.30	0.30	0.30	0.30
Sodium alginate	2.00	2.00	2.00	2.00	2.00	2.00
BHT	0.20	0.20	0.20	0.20	0.20	0.20
Cellulose	0.50	0.475	0.45	0.425	0.40	0.30
Astaxanthin ^3^ (10%)	0.00	0.025	0.05	0.075	0.10	0.20
Proximate composition (%)						
Dry matter	95.33	95.02	95.19	94.58	95.19	95.09
Crude protein	46.05	46.25	46.35	46.28	46.45	46.64
Crude lipid	10.05	10.42	10.37	10.32	10.72	10.30
Ash	9.73	9.75	9.85	9.62	9.80	9.91
Astaxanthin (mg/kg)	0.00	24.20	45.80	72.40	94.20	195.00

^1, 2^ Vitamin and Mineral premix were prepared from Xie et al. [22]. ^3^ Purchased from DSM company, Shanghai, China.

**Table 2:** Fatty acid composition in hepatopancreas of *Portunus trituberculatus* fed with different experimental diets.

Fatty Acids (mg/g)	Dietary Astaxanthin Levels (mg/kg)					
	0.0	24.2	45.8	72.4	94.2	195.0
12:00	0.08 ± 0.01	0.06 ± 0.02	0.07 ± 0.01	0.10 ± 0.02	0.09 ± 0.01	0.05 ± 0.00
14:00	4.36 ± 0.54 ^a^	4.72 ± 0.46 ^b^	4.03 ± 0.52 ^ab^	5.06 ± 0.65 ^b^	5.33 ± 0.08 ^b^	2.79 ± 0.05 ^a^
16:00	41.07 ± 1.43 ^b^	38.70 ± 0.96 ^b^	37.08 ± 1.91 ^b^	40.60 ± 3.03 ^b^	37.28 ± 3.09 ^b^	28.34 ± 0.27 ^a^
18:00	17.28 ± 0.91 ^b^	15.04 ± 0.85 ^b^	15.24 ± 0.62 ^b^	17.31 ± 1.31 ^b^	15.15 ± 1.35 ^b^	11.37 ± 0.44 ^a^
20:00	0.92 ± 0.07 ^c^	0.81 ± 0.03 ^bc^	0.76 ± 0.08 ^bc^	0.79 ± 0.04 ^bc^	0.66 ± 0.09 ^ab^	0.47 ± 0.03 ^a^
∑SFA ^1^	63.71 ± 2.95 ^b^	59.35 ± 2.3 ^b^	57.18 ± 2.9 ^b^	63.86 ± 4.79 ^b^	57.84 ± 5.19 ^b^	43.02 ± 0.75 ^a^
16:1n-7	7.68 ± 0.81 ^b^	7.99 ± 0.80 ^b^	8.24 ± 0.50 ^b^	7.65 ± 0.08 ^b^	9.17 ± 0.40 ^b^	4.99 ± 0.33 ^a^
18:1n-9	75.41 ± 4.09 ^b^	66.24 ± 0.09 ^b^	66.89 ± 3.53 ^b^	75.87 ± 5.88 ^b^	66.77 ± 5.46 ^b^	48.28 ± 1.22 ^a^
20:1n-9	3.20 ± 0.31 ^b^	2.41 ± 0.09 ^ab^	2.70 ± 0.27 ^b^	2.81 ± 0.32 ^b^	2.50 ± 0.21 ^b^	1.67 ± 0.06 ^a^
22:1n-11	0.56 ± 0.03 ^c^	0.42 ± 0.02 ^bc^	0.44 ± 0.00 ^bc^	0.50 ± 0.07 ^bc^	0.38 ± 0.06 ^ab^	0.26 ± 0.02 ^a^
∑MUFA ^2^	86.86 ± 5.23 ^b^	77.07 ± 0.60 ^b^	77.28 ± 4.37 ^b^	88.29 ± 7.64 ^b^	77.62 ± 6.93 ^b^	55.20 ± 1.54 ^a^
18:2n-6 (LA)	24.85 ± 1.77 ^b^	25.30 ± 0.85 ^b^	23.89 ± 0.66 ^b^	25.95 ± 1.97 ^b^	26.35 ± 1.10 ^b^	16.71 ± 0.36 ^a^
18:3n-6	0.09 ± 0.01 ^ab^	0.11 ± 0.00 ^b^	0.08 ± 0.01 ^ab^	0.11 ± 0.02 ^b^	0.12 ± 0.01 ^b^	0.06 ± 0.01 ^a^
20:2n-6	6.42 ± 0.21 ^d^	4.78 ± 0.02 ^b^	5.64 ± 0.33 ^c^	5.29 ± 0.36 ^bc^	5.46 ± 0.05 ^bc^	4.00 ± 0.05 ^a^
20:4n-6 (ARA)	2.58 ± 0.09 ^cd^	2.78 ± 0.16 ^d^	2.37 ± 0.08 ^bc^	2.70 ± 0.18 ^cd^	2.08 ± 0.05 ^ab^	1.89 ± 0.01 ^a^
∑n-6 PUFA ^3^	34.49 ± 2.29 ^b^	32.87 ± 1.01 ^b^	31.15 ± 1.36 ^b^	35.18 ± 2.70 ^b^	31.70 ± 2.43 ^b^	22.67 ± 0.39 ^a^
18:3n-3 (ALA)	3.99 ± 0.15 ^b^	4.09 ± 0.39 ^b^	3.23 ± 0.50 ^ab^	4.03 ± 0.56 ^b^	4.45 ± 0.21 ^b^	2.26 ± 0.10 ^a^
18:4n-3	0.45 ± 0.01 ^b^	0.50 ± 0.08 ^b^	0.41 ± 0.04 ^b^	0.45 ± 0.05 ^b^	0.53 ± 0.05 ^b^	0.24 ± 0.01 ^a^
20:4n3	0.37 ± 0.04	0.41 ± 0.06	0.42 ± 0.08	0.59 ± 0.09	0.51 ± 0.17	0.37 ± 0.04
20:5n-3 (EPA)	17.93 ± 1.11 ^b^	16.75 ± 0.51 ^b^	16.17 ± 1.00 ^b^	18.27 ± 1.76 ^b^	18.48 ± 0.84 ^b^	11.68 ± 0.48 ^a^
22:5n-3	2.61 ± 0.24 ^b^	2.42 ± 0.11 ^b^	2.38 ± 0.14 ^b^	2.88 ± 0.26 ^b^	2.89 ± 0.08 ^b^	1.75 ± 0.04 ^a^
22:6n-3 (DHA)	10.31 ± 0.61 ^ab^	11.67 ± 0.80 ^bc^	11.12 ± 0.40 ^bc^	12.52 ± 1.10 ^c^	12.57 ± 0.52 ^c^	8.67 ± 0.11 ^a^
∑n-3 PUFA ^4^	35.69 ± 2.62 ^b^	36.34 ± 2.24 ^b^	33.26 ± 2.32 ^ab^	38.10 ± 4.03 ^b^	40.24 ± 1.52 ^b^	24.97 ± 0.69 ^a^
DHA/EPA	1.66 ± 0.03 ^b^	1.51 ± 0.06 ^b^	1.52 ± 0.07 ^b^	1.55 ± 0.07 ^b^	1.39 ± 0.03 ^a^	1.35 ± 0.04 ^a^
∑TFA ^5^	220.75 ± 12.95 ^b^	205.63 ± 6.15 ^b^	198.87 ± 10.63 ^b^	225.43 ± 19.10 ^b^	203.39 ± 18.64 ^b^	145.86 ± 3.10 ^a^

^1^ SFA, saturated fatty acid: 16:0, 14:0, 18:0, 17:0, 20:0, 21:0, 22:0; ^2^ MUFA, monounsaturated fatty acids: 16:1 n, 18:1n-9, 20:1n-9, 22:1n-11; ^3^ n-6 PUFA: 18:2n-6, 20:2n-6, 20:4n-6, 22:4n-6; ^4^ n-3 PUFA: 18:3n-3, 18:4n-3, 20:4n-3, 20:5n-3, 22:5n-3, 22:6n-3; ^5^ TFA, total fatty acids. Values were represented as the means of three replications. Means in the same row with different superscripts are significantly different (*p* < 0.05).

## Data Availability

The data that support the findings of this study are available on request from the corresponding author.

## Supplementary Materials

The following supporting information can be downloaded at: https://www.mdpi.com/article/10.3390/antiox13050522/s1, Table S1: Primers used in this study.

## References

[1] Yu W., Liu J. (2020). Astaxanthin isomers: Selective distribution and isomerization in aquatic animals. Aquaculture.

[2] Li B., Lee J.Y., Luo Y. (2023). Health benefits of astaxanthin and its encapsulation for improving bioavailability: A review. J. Agric. Food Res..

[3] Najoan G.C., Prasetyaningsih A., Prakasita V.C., Wisaksono A.A., Rahardjo D. (2021). Anti-inflammatory activity test of astaxan thin extract from *Litopenaeus vannamei* shrimp waste against the number of neutrophils and lymphocytes in white rats (*Rattus norvegicus*) injected with carrageenin. Sch. Acad. Biosci..

[4] McGraw K.J., Toomey M.B. (2010). Carotenoid accumulation in the tissues of zebra finches: Predictors of integument tary pigmentation and implications for carotenoid allocation strategies. Physiol. Biochem. Zool. PBZ.

[5] Yao Q., Ma J., Chen X., Zhao G., Zang J. (2023). A natural strategy for astaxanthin stabilization and color regulation: Interaction with proteins. Food Chem..

[6] Gowd V., Xiao J., Wang M., Chen F., Cheng K. (2021). Multi-mechanistic antidiabetic potential of astaxanthin: An update on preclinical and clinical evidence. Mol. Nutr. Food Res..

[7] Wang S., Qi X. (2022). The putative role of astaxanthin in neuroinflammation modulation: Mechanisms and therapeutic potential. Front. Pharmacol..

[8] Deng X., Wang M., Hu S., Feng Y., Shao Y., Xie Y., Wu M., Chen Y., Shi X. (2019). The neuroprotective effect of astaxanthin on pilocarpine-Induced status epilepticus in rats. Front. Cell Neurosci..

[9] Niu T., Xuan R., Jiang L., Wu W., Zhen Z., Song Y., Hong L., Zheng K., Zhang J., Xu Q. (2018). Astaxanthin induces the Nrf2/HO–1 antioxidant pathway in human umbilical vein endothelial cells by generating trace amounts of ROS. J. Agric. Food Chem..

[10] Zarneshan S.N., Fakhri S., Farzaei M.H., Khan H., Saso L. (2020). Astaxanthin targets *PI3K*/*Akt* signaling pathway toward po tential therapeutic applications. Food Chem. Toxicol..

[11] Long X., Wu X., Zhao L., Liu J., Cheng Y. (2017). Effects of dietary supplementation with *Haematococcus pluvialis* cell powder on coloration, ovarian development and antioxidation capacity of adult female Chinese mitten crab, *Eriocheir sinensis*. Aquaculture.

[12] Ma N., Long X., Liu J., Chang G., Deng D., Cheng Y., Wu X. (2019). Defatted *Haematococcus pluvialis* meal can enhance the coloration of adult Chinese mitten crab *Eriocheir sinensis*. Aquaculture.

[13] Wu X., Zhao L., Long X., Liu J., Su F., Cheng Y. (2017). Effects of dietary supplementation of *Haematococcus pluvialis* powder on gonadal development, coloration and antioxidant capacity of adult male Chinese mitten crab (*Eriocheir sinensis*). Aquac. Res..

[14] Zhang Y., Qian C., Huang J., Li J., Jiang X., Li Z., Cheng Y., Li J. (2023). Suitable natural astaxanthin supplementation with *Haematococcus pluvialis* improves the physiological function and stress response to air exposure of juvenile red swamp crayfish (*Procambarus clarkii*). Aquaculture.

[15] Jiang X., Zu L., Wang Z., Cheng Y., Yang Y., Wu X. (2020). Micro-algal astaxanthin could improve the antioxidant capability, immunity and ammonia resistance of juvenile Chinese mitten crab, *Eriocheir sinensis*. Fish. Shellfish Immun..

[16] Lu Y., Zhang J., Cao J., Liu P., Li J., Meng X. (2022). Long-term ammonia toxicity in the hepatopancreas of swimming crab *Portunus trituberculatus*: Cellular stress response and tissue damage. Front. Mar. Sci..

[17] Tsai M.C., Huang S.C., Chang W.T., Chen S.C., Hsu C.L. (2020). Effect of astaxanthin on the inhibition of lipid accumulation in 3T3-L1 adipocytes via modulation of lipogenesis and fatty acid transport Pathways. Molecules.

[18] Sun P., Jin M., Jiao L., Monroig Ó., Navarro J.C., Tocher D.R., Betancor M.B., Wang X., Yuan Y., Zhou Q. (2020). Effects of dietary lipid level on growth, fatty acid profiles, antioxidant capacity and expression of genes involved in lipid metabolism in juvenile swimming crab, *Portunus trituberculatus*. Br. J. Nutr..

[19] Xie S., Yin P., Tian L., Yu Y., Liu Y., Niu J. (2020). Dietary supplementation of astaxanthin improved the growth performance, antioxidant ability and immune response of juvenile largemouth bass (*Micropterus salmoides*) fed high-fat diet. Marine Drugs.

[20] Liao Z., Xu H., Wei Y., Zhang Q., Liang M. (2018). Dietary astaxanthin differentially affected the lipid accumulation in the liver and muscle of the marine teleost, tiger puffer *Takifugu rubripes*. Aquac. Res..

[21] Ritola O., Tossavainen K., Kiuru T., Lindstrom-Seppa P., Molsa H. (2002). Effects of continuous and episodic hyperoxia on stress and hepatic glutathione levels in one-summer-old rainbow trout (*Oncorhynchus mykiss*). J. Appl. Ichthyol..

[22] Xie S., Li X., Yang Y., Guo C., Zhang X., Zhu T., Luo J., Yang Z., Zhao W., Cui Y. (2023). Effects of dietary isoleucine level on growth and expression of genes related to nutritional and physiological metabolism of swimming crab (*Portunus trituberculatus*). Aquaculture.

[23] Luo J., Zhu T., Wang X., Cheng X., Yuan Y., Jin M., Betancor M.B., Tocher D.R., Zhou Q. (2020). Toxicological mechanism of excessive copper supplementation: Effects on coloration, copper bioaccumulation and oxidation resistance in mud crab *Scylla paramamosain*. J. Hazard. Mater..

[24] Jin M., Monroig Ó., Lu Y., Yuan Y., Li Y., Ding L., Tocher D.R., Zhou Q. (2017). Dietary DHA/EPA ratio affected tissue fatty acid profiles, antioxidant capacity, hematological characteristics and expression of lipid-related genes but not growth in juvenile black seabream (*Acanthopagrus schlegelii*). PLoS ONE.

[25] Association of Official Analytical Chemists Official (AOAC) (1995). Methods of Analysis.

[26] Yang Z., Guo C., Xie S., Zhang Y., Zhu T., Zhao W., Luo J., Jin M., Zhou Q. (2022). Interactive effects of dietary cholesterol and phospholipids on growth and metabolism of juvenile swimming crab, *Portunus trituberculatus*. Anim. Feed. Sci. Technol..

[27] Livak K.J., Schmittgen T.D. (2001). Analysis of relative gene expression data using real-time quantitative PCR and the 2^−ΔΔCT^ method. Methods.

[28] Su F., Yu W., Liu J. (2020). Comparison of effect of dietary supplementation with *Haematococcus pluvialis* powder and synthetic astaxanthin on carotenoid composition, concentration, esterification degree and astaxanthin isomers in ovaries, hepatopancreas, carapace, epithelium of adult female Chinese mitten crab (*Eriocheir sinensis*). Aquaculture.

[29] Lim K.C., Yusoff F.M., Shariff M., Kamarudin M.S. (2018). Astaxanthin as feed supplement in aquatic animals. Rev. Aquac..

[30] Chien Y.H., Pan C.H., Hunter B. (2003). The resistance to physical stresses by Penaeus monodon juveniles fed diets supplemented with astaxanthin. Aquaculture.

[31] Han T., Li X., Wang J., Wang C., Yang M., Zheng P. (2018). Effects of dietary astaxanthin (AX) supplementation on pigmentation, antioxidant capacity and nutritional value of swimming crab, *Portunus trituberculatus*. Aquaculture.

[32] Wade N.M., Cheers S., Bourne N., Irvin S., Blyth D., Glencross B.D. (2017). Dietary astaxanthin levels affect colour, growth, carotenoid digestibility and the accumulation of specific carotenoid esters in the giant tiger shrimp, *Penaeus monodon*. Aquac. Res..

[33] Daly B., Swingle J.S., Eckert G.L. (2013). Dietary astaxanthin supplementation for hatchery-cultured red king crab, *Paralithodes camtschaticus*, juveniles. Aquac. Nutr..

[34] Doolan B.J., Booth M.A., Allan G.L., Jones P.L. (2008). Effects of dietary astaxanthin concentration and feeding period on the skin pigmentation of Australian snapper *Pagrus auratus* (Bloch & Schneider, 1801). Aquac. Res.

[35] Yi X., Xu W., Zhou H., Zhang Y., Luo Y., Zhang W., Mai K. (2014). Effects of dietary astaxanthin and xanthophylls on the growth and skin pigmentation of large yellow croaker *Larimichthys croceus*. Aquaculture.

[36] Naguib Y.M.A. (2000). Antioxidant activities of astaxanthin and related carotenoids. J. Agric. Food Chem..

[37] Shimidzu N., Goto M., Miki W. (1996). Carotenoids as singlet oxygen quenchers in marine organisms. Fish. Sci..

[38] Niu J., Tian L.X., Liu Y.J., Yang H.J., Ye C.X., Gao W., Mai K.S. (2009). Effect of dietary astaxanthin on growth, survival, and stress tolerance of postlarval shrimp, *Litopenaeus vannamei*. J. World Aquac. Soc..

[39] Lei X.G., Zhu J.-H., Cheng W.-H., Bao Y., Ho Y.-S., Reddi A.R., Holmgren A., Arnér E.S.J. (2016). Paradoxical Roles of Antioxidant Enzymes: Basic Mechanisms and Health Implications. Physiol. Rev..

[40] Moren M., Opstad I., Berntssen M., Infante J.-L.Z., Hamre K. (2004). An optimum level of vitamin A supplements for Atlantic halibut (*Hippoglossus hippoglossus* L.) juveniles. Aquaculture.

[41] Hernandez L., Teshima S.-I., Ishikawa M., ALAM S., Koshio S., Tanaka Y. (2005). Dietary vitamin A requirements of juvenile Japanese flounder *Paralichthys olivaceus*. Aquac. Nutr..

[42] Huang Q., Wang X., Bu X., Song Y., You J., Zhang C., Qin C., Qin J., Chen L. (2022). Dietary vitamin A affects growth performance, immunity, antioxidant capacity, and lipid metabolism of juvenile Chinese mitten crab *Eriocheir sinensis*. Aquaculture.

[43] Zhang J., Liu Y.J., Tian L.X., Yang H.J., Liang G.Y., Yue Y.R., Xu D.H. (2013). Effects of dietary astaxanthin on growth, antiox idant capacity and gene expression in Pacific white shrimp *Litopenaeus vannamei*. Aquac. Nutr..

[44] Behringer D.C. (2012). Diseases of wild and cultured juvenile crustaceans: Insights from below the minimum landing size. J. Invertebr. Pathol..

[45] Chang M.X., Xiong F. (2020). Astaxanthin and its effects in inflammatory responses and inflammation-associated diseases: Recent advances and future directions. Molecules.

[46] Muta T., Iwanaga S. (1996). The role of hemolymph coagulation in innate immunity. Curr. Opin. Immunol..

[47] Xue Q., Renault T. (2000). Enzymatic activities in european flat oyster, ostrea edulis, and Pacific oyster, crassostrea gigas, hemlymph. J. Invertebr. Pathol..

[48] Wu J.Y., Lai Y.C., Chang C.L., Hung W.C., Wu H.M., Liao Y.C., Huang C.H., Liu W.R. (2018). Facile and green synthesis of graphene-based conductive adhesives via liquid exfoliation process. Nanomaterials.

[49] Jiang M., Zhao H.H., Zai S.W., Shepherd B., Wen H., Deng D.F. (2019). A defatted microalgae meal (*Haematococcus pluvialis*) as a partial protein source to replace fishmeal for feeding juvenile yellow perch *Perca flavescens*. J. Appl. Phycol..

[50] Cheng Y., Wu S. (2019). Effect of dietary astaxanthin on the growth performance and nonspecific immunity of red swamp crayfish *Procambarus clarkii*. Aquaculture.

[51] Leone A.M., Palmer R.M., Knowles R.G., Francis P.L., Ashton D.S., Moncada S. (1991). Constitutive and inducible nitric oxide synthases incorporate molecular oxygen into both nitric oxide and citrulline. J. Biol. Chem..

[52] Chakravortty D., Hensel M. (2003). Inducible nitric oxide synthase and control of intracellular bacterial pathogens. Microbes Infect..

[53] Jiang G., Yu R., Zhou M. (2006). Studies on nitric oxide synthase activity in haemocytes of shrimps *Fenneropenaeus chinensis* and *Marsupenaeus japonicus* after white spot syndrome virus infection. Nitric. Oxide..

[54] Verma R., Balakrishnan L., Sharma K., Khan A.A., Advani J., Gowda H., Tripathy S.P., Suar M., Pandey A., Gandotra S. (2016). A network map of Interleukin-10 signaling pathway. J. Cell Commun. Signal.

[55] He Q., Xiao S., Zhang C., Zhang Y., Shi H., Zhang H., Lin F., Liu X., Yang H., Wang Q. (2021). Modulation of the growth performance, biochemical parameters, and non-specific immune responses of the hybrid grouper (*Epinephelus fuscoguttatus*♀ × *E*. *lanceolatus*♂) by two kinds of Chinese herb. Aquac. Rep..

[56] Yin B., Liu H., Tan B., Dong X., Chi S., Yang Q., Zhang S., Chen L. (2018). Cottonseed protein concentrate (CPC) suppresses immune function in different intestinal segments of hybrid grouper ♀*Epinephelus fuscoguttatus* × ♂*Epinephelus lanceolatu* via TLR-2/MyD88 signaling pathways. Fish. Shellfish Immun..

[57] Huang Y., Chen Y.H., Wang Z., Wang W., Ren Q. (2014). Novel myeloid differentiation factor 88, EsMyD88, exhibits EsTube-binding activity in Chinese mitten crab *Eriocheir sinensis*. Dev. Comp. Immunol..

[58] Li F., Xiang J. (2013). Recent advances in researches on the innate immunity of shrimp in China. Dev. Comp. Immunol..

[59] Moynagh P.N. (2005). TLR signalling and activation of IRFs: Revisiting old friends from the NF-κB pathway. Trends Immunol..

[60] Kanwugu O.N., Glukhareva T.V. (2023). Activation of Nrf2 pathway as a protective mechanism against oxidative stress-induced diseases: Potential of astaxanthin. Arch. Biochem. Biophys..

[61] Zhang W., Feng C., Jiang H. (2021). Novel target for treating Alzheimer’s Diseases: Crosstalk between the Nrf2 pathway and autophagy. Ageing Res. Rev..

[62] Mu C., Zhao J., Wang L., Song L., Song X., Zhang H., Qiu L., Gai Y., Cui Z. (2009). A thioredoxin with antioxidant activity identified from *Eriocheir sinensis*. Fish. Shellfish Immun..

[63] Mu C., Zhao J., Wang L., Song L., Zhang H., Li C., Qiu L., Gai Y. (2009). Molecular cloning and characterization of peroxire doxin 6 from Chinese mitten crab *Eriocheir sinensis*. Fish. Shellfish Immun..

[64] Wang H., Dai A., Liu F., Guan Y. (2015). Effects of dietary astaxanthin on the immune response, resistance to white spot syn drome virus and transcription of antioxidant enzyme genes in Pacific white shrimp *Litopenaeus vannamei*. Iran. J. Fish. Sci..

[65] NRC (2011). Nutrient Requirements of Fish and Shrimp.

[66] Barrento S., Marques A., Teixeira B., Mendes R., Bandarra N., Vaz-Pires P., Nunes M.L. (2010). Chemical composition, cholesterol, fatty acid and amino acid in two populations of brown crab Cancer pagurus: Ecological and human health implications. J. Food Compos. Anal..

[67] Wang Z., Cai C., Cao X., Zhu J., He J., Wu P., Ye Y. (2018). Supplementation of dietary astaxanthin alleviated oxidative damage induced by chronic high pH stress, and enhanced carapace astaxanthin concentration of Chinese mitten crab *Eriocheir sinensis*. Aquaculture.

[68] Wei B., Yang Z., Cheng Y., Wang J., Zhou J. (2018). Effects of the complete replacement of fish oil with linseed oil on growth, fatty acid composition, and protein expression in the Chinese mitten crab (*Eriocheir sinensis*). Proteome Sci..

[69] Yuan Y., Sun P., Jin M., Wang X., Zhou Q. (2019). Regulation of dietary lipid sources on tissue lipid classes and mitochondrial energy metabolism of juvenile swimming crab, *Portunus trituberculatus*. Front. Physiol..

[70] Yuan Y., Xu F., Jin M., Wang X., Hu X., Zhao M., Cheng X., Luo J., Jiao L., Betancor M.B. (2021). Untargeted lipidomics reveals metabolic responses to different dietary n-3 PUFA in juvenile swimming crab (*Portunus trituberculatus*). Food Chem..

[71] Tan S.J., Zhang X., Jin X.K., Li W.W., Li J.Y., Wang Q. (2015). Fatty acid binding protein *FABP3* from Chinese mitten crab *Eriocheir sinensis* participates in antimicrobial responses. Fish. Shellfish Immun..

