# Supplementary material for: Dietary Astaxanthin Can Promote the Growth and Motivate Lipid Metabolism by Improving Antioxidant Properties for Swimming Crab, Portunus trituberculatus
Source: Antioxidants (Basel). 2024 Apr 26;13(5):522. doi: 10.3390/antiox13050522 (PMC11117615; doi:10.3390/antiox13050522)
Supplement: Supplementary file 1 [file antioxidants-13-00522-s001.zip › antioxidants-2957007-supplementary.pdf]

Table S1. Primer pairs sequences used for real-time quantitative PCR.

Gene	Nucletide sequence (from 5' to 3')	Product size (bp)	Accession no.
cMnsod[1]	F: GATCCATCACACCAAGCACC	193	FJ031018.1
	R: GTTCTCCTCCAGCTTCAGGT		
cat[2]	F: GCTCTGACCCTGACTATGCT	180	FJ152102.2
	R: CGACCAACAGGAATTAGCGG		
gsh-px[3]	F: TACCGTTCAGCAAGTACCGT	171	KY216076.1
	R: GTGGTGTTTTCCTGGTGACC		
trx[4]	F: GAAAGGGCGAGAAGGTCCA	188	OP081649
	R: CTACTCCCCAATGAGCCGAT		
tlr4[5]	F: ACTTTTGTGTTGTGCTGCGG	126	MF124331.1
	R: CACCCTCACTCTATGCCCTT		
relish[6]	F: GTGAGTTGAAAATAGTGCGGA	227	MF624027.1
	R: ATTGGTAACTCTGTAGCGTGG		
irak4[7]	F: CCCACACTGCCACTCTCATC	217	AFZ95002.1
	R: TCAGAAGGTTCAGCCTCACTC		
myd88[8]	F: TGCCACTCCCTGGATTACTAT	133	KM521426.2
	R: AGCCAGAGAGTTCCCGTATTC		
hsp70[9]	F: CGTGCAGTAGGCATCGATCT	128	XM_045252351.1
	R: GTGTCTGTGAAGGCCACGTA		
hsp90[10]	F: TGCTGGCCGTATCTACAGAATG	127	XM_045250701.1
	R: GCGGGAAGTGTCCTCTTCAT		
po[11]	F: TTTGCCGAGACAAGCAGTGTG	139	XM_045278589.1
	R: TAGGCAGGCGCAGATGTCTC		
ampk[12]	F: GCTGATGCCAGTGCCATGTA	150	XM_045277426.1
	R: AATGCCAAGATGAGGTGCAAT		
foxo[13]	F: CATAAGTTCTCGCCAGCCTC	108	XM_045253728.1
	R: TAACCTTCAGGACACGGGAG		
pi3k[14]	F: AGCCACCACTCGCTGAACA	124	ADE44090
	R: GGGATGGGACTCTGCTGAAG		
akt[15]	F: GGACTACGAGGCACCAAGAA	179	XM_045254065.1
	R: TGGACCACTT·CATCACGCTC		
nadph[16]	F: CTACTATGCCAGTCGCCGTG	183	XM_045258166.1
	R: TGTGGCCCAAAGTTGATGGT		
fabp1[17]	F: CACTCGCCAGTAGTCAATAGG	219	KU950355
	R: TCACTTAGAGAGCAAAGGTCAC		
fabp3[18]	F: GAAGGCACTTGGTGTTGGA	119	PRJNA432636

	R: TCTTGAGGGTGGAGATGGT		
fatp4[19]	F: AAGAATGACCCAATGCGTG	178	PRJNA432636
	R: GCCAGCGAAAGGTGTCTC		
acox2[20]	F: ACCAACCACGGCATTCAC	116	PRJNA432636
	R: TTGTCCACCCCATTCAGC		
gst[21]	F: CTCCTTGCCCTTGAGTGGAC	163	XM_045273299.1
	R: CTGTGCGGTGTGCTCCTTT		
β-actin	F: CGAAACCTTCAACACTCCCG	155	FJ641977.1
	R: GGATAGCGTGAGGAAGGGCATA		
18s rRNA	F: CGCCTGTTTATCAAAAACAT	142	XM_045249033.1
	R: CCGGTCTGAACTCAGATCACG		
gapdh[22]	F: TGAGGTGAAGGTAGAGGAT	168	XM_045278068.1
	R: CCAGTGAAGTGAGCAGAG		

[1] *cMnsod*, superoxide dismutase; [2] *cat*, catalase; [3] *gsh-px,* glutathione peroxidase; [4] *trx*, thioredoxin; [5] *tlr4*, toll like receptor 4; [6] *relish*, target of rapamycin; [7] *irak4*, interleukin 1 receptor associated kinase 4; [8] *myd88*, myeloid differentiation factor 88; [9] *hsp70*, heat shock protein 70; [10] *hsp90*, heat shock protein 90; [11] *po*, phenol oxidase; [12] *ampk*, Adenosine 5'-monophosphate (AMP)-activated protein kinase α2; [13] *foxo*, forkhead box protein O1; [14] *pi3k*, Phosphoinositide 3-kinase; [15] *akt*, protein kinase B; [16] *nadph*, nicotinamide adenine dinucleotide phosphate; [17] *fabp1*, fatty acid binding protein 1; [18] *fabp3*, fatty acid binding protein 3; [19] *fatp4*, fatty acid transport protein 4; [20] *acox2*, acyl-coenzyme a oxidase 2; [21] *gst*, Glutathione S-transferase; [22] *gapdh*, glyceraldehyde-3-phosphate dehydrogenase.
